# Benchmark datasets for predictive maintenance challenges in steel manufacturing

**DOI:** 10.3389/frai.2026.1770922

**Published:** 2026-04-01

**Authors:** Jakub Jakubowski, Szymon Bobek, Grzegorz J. Nalepa

**Affiliations:** 1 Department of Applied Computer Science, AGH University of Science and Technology, Kraków, Poland; 2 Department of Human-Centered Artificial Intelligence, Institute of Applied Computer Science, Faculty of Physics, Astronomy and Applied Computer Science, Jagiellonian University, Kraków, Poland

**Keywords:** anomaly detection, cold rolling, data, machine learning, predictive maintenance

## 1 Introduction

Predictive Maintenance (PdM) is a strategy for keeping the good condition of industrial machinery, which utilizes the advanced data analytics to predict equipment malfunctions as soon as possible. The objectives of PdM include minimizing downtime, reducing operational costs, and ensuring high product quality. These methods can be widely adopted across various industries, including steel production, which is a crucial industry that supplies materials for sectors such as construction, automotive, energy, and manufacturing. One of the integral steps of steel production process is cold rolling, which aims to reduce the thickness of hot-rolled steel. Like in any other industrial process, presence of anomalies or failures during cold rolling, may lead to unexpected downtime, quality losses or reduced performance.

One of the most important fields related to the development of PdM methods is Machine Learning (ML). ML provides tools and techniques to analyze large volumes of data from industrial processes, enabling the identification of patterns related with normal working conditions and degradation. The development of ML methods in industrial settings, including tandem cold mills (TCM), is often hampered by a lack of high-quality datasets. Most research on PdM in the steel industry depends on proprietary, on-site data that tends to be unlabeled, and unavailable to the wider community, hindering reproducibility and cross-study comparison ((Jakubowski et al., 2024b)). Furthermore, the absence of publicly available industrial data from TCM makes it impossible to benchmark different methods against each other, hindering the clear advancement of PdM methods development. To address these issues, we have created synthetic datasets from the cold rolling process aimed at identifying anomalies with a physical basis. These datasets can be used to support the development and evaluation of various diagnostic tasks, including anomaly detection, fault detection, and fault diagnosis.

**Figure 1:**
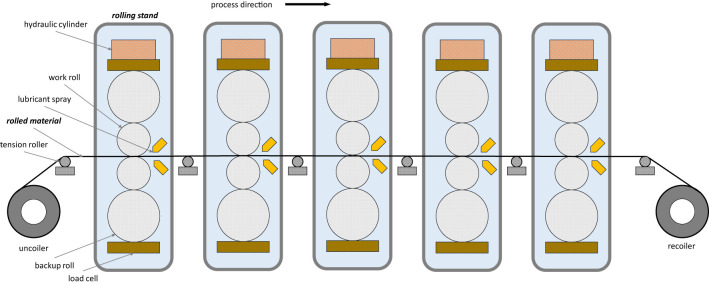
Schematic diagram of 5-stand tandem cold mill.

Figure 1 presents a schematic overview of a TCM consisting of five rolling stands. The production process begins with uncoiling the material and feeding it into the mill. As the material gradually moves through the rolling stands, it is gripped, and tensions are established to ensure the stability of the strip. At the mill's exit, the strip is recoiled. Once the recoiler grips the strip, the process accelerates to the target speed and concludes when the entire coil has been rolled and dispatched from the production line.

The synthetic datasets were generated using mathematical model of cold rolling process, available in the literature of the domain, which contains methods for calculation of several key process parameters, i.e., rolling force, torque, speed, tension, gap, thickness reduction, and motor power. In conceptualization of this dataset, research into the existing models of cold rolling has been undertaken, to provide a reliable simulation of the process. Based on the predefined product parameters and evolving mill parameters, process variables are calculated. The PdM aspect of the datasets is realized through introduction of anomalies, which are related to a specific kind of failures in the process. In total, we generated six diverse datasets, each varying in complexity, to enable benchmarking of methods across various levels of complexity. Easier datasets are characterized by lower number of anomaly types and smaller number of product types. In more complex datasets, we also introduce data shifts, requiring the development of methods, which adapt to changes in the process. Simpler datasets can serve as a starting point for developing new methods, while the more challenging ones typically require advanced techniques such as concept drift detection and data stream learning.

In summary, these datasets can advance development of PdM methodologies devoted to industrial processes in general, with a focus on the cold rolling process. The synthetic nature of this dataset ensures the existence of ground-truth labels for all observations. This allows for the unbiased comparison of different methods, without need of using poorly labeled data from a real manufacturing process. While this approach allows for the generation of high-quality datasets, it also has its limitations, as the simulated data may not capture all the complexities and nuances of real-world data. In particular, this data may not fully reflect the noise, production variability, and unexpected anomalies that can occur in real manufacturing processes. Similarly to other public datasets available, for example, C-MAPSS (Saxena et al., 2008) or TEP (Chen, 2019), the datasets presented in this work should be treated as a benchmark for the development of new methods, rather than a perfect representation of real-world data. The generation of six different subsets, varying in complexity, enables researchers to verify both simple and complex approaches.

## 2 Methods

The mathematical modeling of cold rolling process is well-studied field, as shown by Alexander (1972); Lenard et al. (1999); Lenard (2014a); Liu and Lee (2005). Models of cold rolling were developed to understand the relationships between the process variables and optimize the rolling process to achieve optimal productivity of actual production sites, as shown in Pires et al. (2006). They typically involve complex equations describing material flow, stress-strain relationships, frictional forces, and deformations. In this work, we have developed such a model, with the aim of simulating the normal and abnormal process behaviors, rather than optimizing the efficiency of actual manufacturing line.

In following subsections, we present the data and methodology used for simulating the process in a TCM. The data needed to simulate the process can be divided into three main groups: (1) product data, (2) mill data, and (3) process variables. The characteristics of the steel product and the parameters of the installation serve as an input to the mathematical model, while the process variables are the output of the model. They all constitute an integral part of the feature space in our datasets.

### 2.1 Product information

The product data includes the dimensions of the materials (entry thickness, exit thickness, and width) and their mechanical properties, which are expressed as stress-strain curves. During cold rolling material undergoes plastic deformation at each rolling stand, resulting in the gradual increase in mechanical properties, such as yield strength. The evolution of yield strength during rolling can be expressed as a function of strain and approximated by the following equation from Hlavacek (1968):


$$\begin{array}{l} \sigma =K\varepsilon^{n} \end{array}$$ (1)

where σ is yield strength, *K* is the modulus of plasticity and *n* the hardening exponent. These are material properties, which can be assumed to be constant for a specific material grade. The total true strain ε of the material is calculated using equation:


$$\begin{array}{l} \varepsilon =\ln\frac{h_{in}}{h_{out}} \end{array}$$ (2)

where *h*_*in*_ is the input thickness and *h*_*out*_ is the output thickness.

### 2.2 Mill characteristics

The configuration of the mill plays a crucial role in modeling the cold rolling process. Factors such as the arrangement of rolls, their size, and the power of the machinery determine the equipment setup, including production speed and load distribution. The main parameters that characterize a TCM include:

- Number of the rolling stands—higher number of stands allows for greater total reduction and smaller load at individual stands.
- Characteristics of the motors—they decide the maximum power and speed, which can be achieved during production process, thus influencing production capacity.
- Lubrication parameters—they affect the friction in a rolling gaps and cooling process, impacting both roll wear and the quality of the product.
- Process limits—they determine minimum and maximum values of process variables.

### 2.3 Mathematical modeling of rolling process

**Figure 2:**
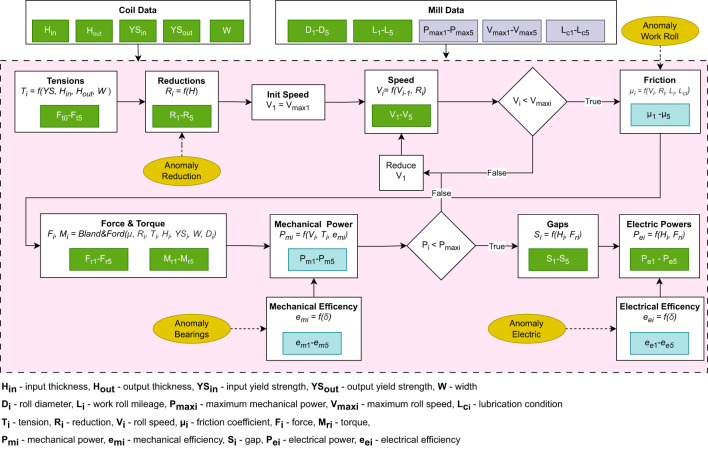
The overview of the mathematical simulation, which we used to generate datasets from cold rolling process. H_*in*_, input thickness; H_*out*_, output thickness; YS_*in*_-input yield strength; YS_*out*_, output yield strength; W, width; D_*i*_, roll diameter; L_*i*_, work roll mileage; P_*maxi*_, maximum mechanical power; V_*maxi*_, maximum roll speed; L_*ci*_, lubrication condition; T_*i*_, tension; R_*i*_, reduction; V_*i*_, roll speed; μ_*i*_, friction coefficient; F_*i*_, force; M_*ri*_, torque; P_*mi*_, mechanical power; e_*mi*_, mechanical efficiency; S_*i*_, gap; P_*ei*_, electrical power; e_*ei*_, electrical efficiency.

Given the product information and mill configuration, the observed variables in the rolling mill are determined using mathematical equations that describe the cold rolling process. A high-level overview of the simulation process is presented in Figure 2, which summarizes the main calculation steps that lead to the generation of a single observation in the dataset. The features colored in green are observed (included in the dataset), while those colored in gray are only intermediate features, which are not recorded.

Given the product information and mill configuration, the observed variables in the rolling mill are determined using mathematical equations that describe the cold rolling process. In the first step, the model determines the strip tension (based on the yield stress and cross-sectional area) and reduction schedule [using approach proposed by Bryant et al. (1972)].


$$\begin{array}{l} \Delta h_{i}=\left(h_{in}-h_{out}\right)\frac{2c_{a}}{i} \end{array}$$ (3)

where *h*_*i*_ represents the strip thickness after passing through stand *i*, *h*_*in*_, and *h*_*out*_ denote the input and output product thicknesses respectively, and *c*_*a*_ stands for the adjustment coefficient. The thickness after each stand is determined based on the calculated change in thickness. Typically, the resultant output thickness differs from the desired thickness. Therefore, the values obtained are adjusted using the following equation:


$$\begin{array}{l} h_{fi}=h_{i}\frac{h_{out}}{h_{5}} \end{array}$$ (4)

where *h*_*fi*_ represents the final thickness after each stand, *h*_*i*_ denotes the thickness calculated in the preceding step, and *h*_5_ signifies the thickness at the last stand calculated in the preceding step. The thickness of the product before and after each stand, determine the required reduction.

Once thickness is set, the rolling simulation must compute forces and torques, which require applying plasticity theory and rolling mechanics. Some well-known analytical models that meet these criteria include those by Orowan (1943), Bland and Ford (1948) and Alexander (1972). In our simulation, we utilized the model developed by Bland and Ford (1948).

It uses geometrical and physical relationships between the steel and work roll parameters together with several assumptions to compute the approximate force and torque to roll a given material. For example, an equation describing the rolling force is as follows:


$$\begin{array}{l} P_{r}=2R^{\prime }\int_{0}^{\phi_{n}}\frac{kh}{h_{o}}\left(1-\frac{\sigma_{o}}{k_{o}}\right)\exp\left(\mu H\right)d\phi \\ \;+\;2R^{\prime }\int_{\phi_{n}}^{\phi_{o}}\frac{kh}{h_{i}}\left(1-\frac{\sigma_{i}}{k_{i}}\right)\exp\left(\mu \left(H_{i}-H\right)\right)d\phi \end{array}$$ (5)

where *P*_*r*_—rolling force [N]; *R*′—deformed roll radius [m]; ϕ—contact angle [rad]; *k*—strip yield stress [Pa]; σ—strip tension [Pa]; *h*—strip thickness [m]; μ—friction coefficient [-]; *H*—dimensionless thickness [-]; *i*—entry; *o*—exit, *n*—neutral point.

For a detailed description of the calculation procedure, we refer to original paper by Bland and Ford (1948).

A key factor in accurately calculating roll force and torque is the friction coefficient in the roll gap. Although friction is difficult to determine precisely in practice, many studies have examined factors affecting it, with Lenard (2014b) providing a notable summary. We build on this work and simulate friction coefficient as a function of roll speed, work roll mileage, lubrication mileage and reduction.

Before running the analytical model that calculates forces and torques, an initial speed distribution is determined, based on the predefined stand limits and reduction schedule, so that friction coefficient can be estimated. Except for that, analytical model considers the entry and exit thickness, strip width, tensions, yield strength of the material, and diameter of work rolls. With rolling force and torque known, the mechanical power required—based on rolling speed, roll diameter, and torque—can be computed using basic dynamics as presented in Hanoglu and Šarler (2009):


$$\begin{array}{l} P_{mi}=M_{ri}\frac{v_{i}}{0.5D_{i}} \end{array}$$ (6)

where *P*_*mi*_ is the mechanical power of stand *i*, *M*_*ri*_ is the required rolling torque, *v*_*i*_ is the rolling speed and *D*_*i*_ is the diameter of work rolls. In case of exceeding any of the power limits defined in mill configuration, the rolling speed must be decreased, friction recalculated, and analytical model run once again.

The next step is to determine the size of the rolling gap. This is achieved using mill stretch formula (Lenard, 2014a), which is a function of material thickness in the stand and rolling force (Equations 1–7).


$$\begin{array}{l} s_{i}=h_{i}-\frac{F_{ri}}{S_{i}} \end{array}$$ (7)

where *s*_*i*_ is the roll gap, *h*_*i*_ is the exit thickness, *F*_*ri*_ is the rolling force and *S*_*i*_ is the mill stiffness, which is defined in mill configuration.

Finally, we calculate the electric power consumption, which is determined by dividing mechanical power by the electric efficiency. This ends the single run of the model and produces one observation in a dataset, containing 51 features. Additionally, each record has 16 anomaly labels associated with it, which determine occurrence of anomalies. These are binary labels, in which TRUE indicates the occurrence of a given anomaly type.

After each iteration, mill parameters such as work roll mileage, lubricant condition are updated. If the work rolls mileage or lubricant condition exceeds predefined limits, indicating their wear, they are “replaced,” slightly changing the normal working conditions. The simulation runs in a loop, randomly selecting the next product and its number of repetitions, until the desired size of dataset is obtained.

### 2.4 Anomalies

The primary goal of the datasets is to identify anomalies within the simulated process. We introduced four distinct types of anomalies, which impact the simulation at specific stages of the calculation procedure. The anoamalies are designed in a manner that makes understanding their sources straightforward, making the evaluation of results easier. They are introduced by modifying the parameters of the mathematical model, which results in changes in the process variables, and thus the final product parameters. Below we provide a list of the introduced anomalies with their descriptions.

1. Reduction anomaly—it is introduced by modifying the adjustment coefficient *c*_*a*_ during reduction schedule calculation. This effects in abnormal reduction scheme. The final thickness of the product remains the same.
2. Work roll anomaly—it is defined as increased work roll friction. In practice such phenomenon can happen due to invalid preparation of work rolls or poor lubrication.
3. Bearings anomaly—it is defined as increased rolling torque, which is higher than the one calculated from analytical model. Increased torque can be the effect of high mechanical losses due to e.g., problems with bearings.
4. Electric anomaly—it is related to the decreased electrical efficiency, which effects in increased electrical power of the motor, and indicates problems with the specific motor.

Reduction anomaly is the only anomaly type that affects the entire process, as it modifies the reduction scheme, which is a key factor in determining the thickness after each stand, and thus all subsequent calculations. The other three types of anomalies are specific to individual stands, meaning that they only affect the parameters related to a given stand, and the parameters determined after it. Thus, we have one anomaly label for reduction anomaly, and five labels for each of the other three types of anomalies, indicating their occurrence at each of the five stands.

### 2.5 Data shifts

In a real-world industrial problems, we often deal with the issue of changing normal process conditions. These changes, which are referred to as data shift present a challenge in the development of PdM methods. These shifts in data distribution can occur due to a range of factors such as machinery wear, changes in raw materials, or modifications to manufacturing processes. To enforce development of robust algorithms and methods, which can address this issue, we introduced two types of data shift in our simulation.

Firstly, we simulate the introduction of new, previously unseen products that are rolled in TCM. A poor anomaly detection method will inevitably classify all new products as anomalies since such observations were absent from the dataset before. Conversely, a robust method will effectively generalize to predict the normal behavior of the process under new conditions or will learn this behavior as new products emerge.

Secondly, we introduce a shift related to increased production capacity, characterized by heightened power and motor speed. In such a scenario, the new process speed and power consumption will deviate from previous normal working conditions. This necessitates adapting the prediction model to accommodate these new conditions while ideally retaining as much knowledge from the process as possible.

## 3 Data records

The datasets are publicly available in a Zenodo repository: Jakubowski et al. (2024a). The repository contains six CSV files, each representing a separate rolling simulation. The datasets vary in product types, anomaly types, and data shifts. Each record describes the processing conditions of one steel coil, including entry/exit thickness, width, and yield strengths. Multiple coils can share the same product type. Table 1 summarizes the datasets.

**Table 1:** Summary of datasets.

Dataset	Observations	Anomalies	Share of anomalies	Features	Anomaly types	Unique products	Data shift
tcm5_dataset_1	20009	1,045	5.2%	51	1	4	FALSE
tcm5_dataset_2	20001	1,035	5.2%	51	1	20	FALSE
tcm5_dataset_3	20003	981	4.9%	51	4 (16)	4	FALSE
tcm5_dataset_4	20001	925	4.6%	51	4 (16)	20	FALSE
tcm5_dataset_5	20005	1,031	5.2%	51	4 (16)	5	TRUE
tcm5_dataset_6	20008	954	4.8%	51	4 (16)	25	TRUE

Table 2 summarizes the features present in the dataset and provides their brief description. The “Suffix” column indicates that certain feature is recorded per each stand, meaning five repetitions of the same physical property. The only exception is the “Tension” feature, which occurs six times.

**Table 2:** Description of features.

Feature	Suffixes	Unit	Description
Line no.	–	–	No. observation
Thickness_entry	–	mm	Product entry thickness
Thickness_exit	–	mm	Product exit thickness
Width	–	mm	Product width
ys_entry	–	MPa	Product entry yield strength
ys_exit	–	MPa	Product exit yield strength
Work_roll_diam	1–5	mm	Work roll diameter (stands 1–5)
Work_roll_mileage	1–5	km	Work roll mileage (stands 1–5)
Reduction	1–5	–	Thickness reduction (stands 1–5)
Tension	0–5	N	Tension (0—before stand 1, 1–5—after stand 1–5)
Roll_speed	1–5	–	Linear work roll speed (stands 1–5)
Force	1–5	N	Rolling force (stands 1–5)
Torque	1–5	Nm	Rolling torque (stands 1–5)
Gap	1–5	mm	Stand gap (stands 1–5)
Motor_power	1–5	kW	Electric motor power (stands 1–5)
Anomaly_reduction	–	–	(label) anomaly in reduction scheme
Anomaly_electric	1–5	–	(label) anomaly in electric motor (stands 1–5)
Anomaly_bearing	1–5	–	(label) anomaly in stand bearing (stands 1–5)
Anomaly_work roll	1–5	–	(label) anomaly in work roll friction (stands 1–5)

## 4 Technical validation

For the technical validation of the datasets, we trained several machine learning models for anomaly detection task on all six datasets. We selected several popular methods for anomaly detection, including Autoencoder (AE), Principal Component Analysis (PCA), Isolation Forest (IForest), LODA, One-Class SVM (OCSVM), Histogram-based Outlier Score (HBOS), and K-means clustering. We also included a Baseline model, which is based a ra Table 3 presents the performance of several machine learning models trained for the anomaly detection task. The results show that while anomalies are generally detectable by these models, their performance is far from perfect—particularly on the more challenging datasets. The best results are typically achieved by the Autoencoder model, especially on datasets without data shifts. Half-space Trees, which is a online learning model and updates continuously with each new observation, performed slightly better than batch learning models on datasets 5 and 6 in terms of F1-score. The overall performance of online learning models remains modest, however a more extensive hyperparameter tuning process could improve their performance. These findings suggest that achieving high predictive accuracy on the datasets requires more sophisticated approaches than standard, off-the-shelf methods, as these methods struggle to generalize well across all datasets, particularly those with data shifts.

**Table 3:** Performance of several ML models on anomaly detection task.

	Dataset	Baseline	AE	PCA	IForest	LODA	OCSVM	HBOS	K-means^a^	HST^a^	OCSVM^a^
1	F1	0.07	0.82	0.29	0.69	0.25	0.56	0.66	0.12	0.35	0.10
	AUCPR	0.06	0.87	0.24	0.79	0.24	0.57	0.75	0.09	0.32	0.06
	AUCROC	0.48	0.97	0.71	0.98	0.79	0.93	0.97	0.54	0.82	0.53
	G-mean	0.40	0.88	0.59	0.80	0.49	0.74	0.78	0.42	0.60	0.45
2	F1	0.06	0.61	0.10	0.32	0.13	0.29	0.16	0.09	0.10	0.06
	AUCPR	0.04	0.64	0.08	0.33	0.11	0.26	0.15	0.05	0.12	0.04
	AUCROC	0.48	0.93	0.61	0.82	0.73	0.82	0.72	0.53	0.64	0.49
	G-mean	0.38	0.74	0.52	0.53	0.37	0.56	0.39	0.46	0.24	0.40
3	F1	0.07	0.77	0.33	0.30	0.47	0.42	0.34	0.13	0.24	0.12
	AUCPR	0.05	0.87	0.32	0.35	0.51	0.45	0.41	0.08	0.20	0.06
	AUCROC	0.46	0.97	0.80	0.85	0.92	0.89	0.87	0.58	0.73	0.53
	G-mean	0.35	0.90	0.70	0.55	0.64	0.74	0.52	0.53	0.61	0.39
4	F1	0.08	0.64	0.08	0.13	0.15	0.22	0.10	0.07	0.16	0.08
	AUCPR	0.05	0.68	0.08	0.11	0.09	0.20	0.08	0.06	0.12	0.04
	AUCROC	0.50	0.93	0.64	0.70	0.67	0.82	0.65	0.54	0.63	0.47
	G-mean	0.46	0.77	0.26	0.59	0.41	0.70	0.53	0.31	0.45	0.43
5	F1	0.07	0.20	0.10	0.11	0.10	0.10	0.09	0.09	0.22	0.09
	AUCPR	0.05	0.60	0.13	0.15	0.05	0.09	0.06	0.06	0.25	0.04
	AUCROC	0.49	0.89	0.62	0.63	0.56	0.58	0.51	0.52	0.63	0.48
	G-mean	0.44	0.73	0.52	0.58	0.51	0.53	0.47	0.45	0.58	0.21
6	F1	0.07	0.13	0.10	0.09	0.09	0.10	0.09	0.08	0.14	0.08
	AUCPR	0.04	0.34	0.11	0.11	0.07	0.07	0.08	0.04	0.11	0.04
	AUCROC	0.51	0.80	0.61	0.58	0.56	0.62	0.55	0.52	0.66	0.45
	G-mean	0.41	0.67	0.56	0.54	0.52	0.53	0.47	0.35	0.50	0.32

^a^Online learning models.

The best result for each metric is underlined.

## Data Availability

The datasets presented in this study can be found in online repositories. The names of the repository/repositories and accession number(s) can be found below: https://doi.org/10.5281/zenodo.11469702.
